# Beyond Detection: Conventional and Emerging Biomarkers in Gastrointestinal Cancers

**DOI:** 10.3390/cancers17172725

**Published:** 2025-08-22

**Authors:** Daniel M. Han, Mark R. Wakefield, Yujiang Fang

**Affiliations:** 1 Department of Microbiology, Immunology & Pathology, Des Moines University, West Des Moines, IA 50266, USA; dmhan29@dmu.edu; 2 Department of Surgery, University of Missouri School of Medicine, Columbia, MO 65212, USA; wakefieldmr@health.missouri.edu; 3 Ellis Fischel Cancer Center, University of Missouri School of Medicine, Columbia, MO 65212, USA

**Keywords:** Gastrointestinal cancer, colorectal cancer, gastric cancer, cancer biomarkers, precision medicine, molecular profiling, liquid biopsy, targeted therapy

## Abstract

Gastrointestinal (GI) cancers, particularly colorectal and gastric cancers, majorly contribute to global cancer mortality due to frequent late-stage diagnosis and poor therapeutic response in advanced disease. Earlier detection of GI cancers is needed for a better prognosis. This review examines both traditional and emerging biomarkers that contribute significantly to early detection, prognostication, and prediction of therapeutic resistance or sensitivity. Specifically, we highlight the diagnostic utility of non-invasive liquid biopsy biomarkers such as circulating tumor DNA (ctDNA), microRNAs (miRNAs), and exosomes. Moreover, we discuss the prognostic and predictive value of conventional genetic alterations, including KRAS, BRAF, and HER2. Although new findings have shown the advantages of liquid biopsy over colonoscopy, there are still limitations to the technique, such as cost-effectiveness, technological gaps in low-resource settings, and uncertain detection rates. Further studies are required to test the validity and accessibility of liquid biopsy and its biomarkers in order to advance personalized diagnosis and treatments for GI cancers. Such a study will be helpful for clinicians to better manage patients with GI cancers.

## Simple Summary

Gastrointestinal (GI) cancers, including stomach, colon, and pancreas, are considered one of the most prevalent cancer types, majorly contributing to global cancer mortality. Often, the symptoms GI cancers are non-specific, leading to late diagnosis and detection. To tackle this challenge, scientists and physicians have been searching for “biomarkers.” Biomarkers are signals in blood, tissue, or even cancerous cells that tell us if cancer is present, resistance or sensitivity to certain drugs, and prognosis. This review explains and compares conventional biomarkers and new emerging biomarkers to highlight their benefits and disadvantages.

## 1. Introduction

Gastrointestinal (GI) cancers are cancers in the digestive tract and its associated organs. There were 353,802 new GI cases in the US, accounting for 17.8% of cancer incidents, in 2024. Furthermore, in the same year, 174,320 deaths from GI cancers were reported out of 611,720 total estimated deaths from cancers in the US, representing 28.5% of total estimated deaths [1]. Given its high incidence rate and mortality rates, there is a significant demand for finding and understanding molecular biomarkers for GI cancers for early detection, prognostic evaluation, and targeted therapies to enhance patient outcomes. In 2018, gastric cancer (GC) was the fifth most diagnosed cancer and the third leading cause of cancer-related deaths globally. In comparison, colorectal cancer (CRC) ranked as the third most frequently diagnosed malignancy and the second leading cause of cancer deaths [2].

Two of the most prevalent types of GI cancer, gastric cancer and colorectal cancer, are the focus of this study [3]. Pancreatic cancer, although it is associated with poor prognosis and a high fatality rate, is not the focus of this review. Specifically, this review will cover traditional diagnostic, prognostic, and predictive GI cancer biomarkers, as well as novel biomarkers that have emerged in both the research and clinical fields of oncology.

As technology has developed, there has been a rapid change in the oncology field from traditional organ-specific treatments to personalized and biomarker-guided therapies. This has led to the development of an idea called precision medicine. Precision medicine enables physicians to categorize and populate patients according to their genetic profiles to determine the treatments with the best possible prognosis [4]. This shift allows for more personalized approaches. Additionally, precision medicine is used to prevent patients from taking ineffective treatments (determined according to statistical data analysis) with negative side effects that will do more harm than good. In the GI oncology field, precision medicine plays a significant role in diagnosis, prognosis, and predicting the effectiveness of treatments according to the presence of different types of biomarkers. Previous studies have discovered diagnostic, prognostic, and predictive biomarkers, enabling earlier detection, more accurate prognosis, and tailored therapeutic strategies [5].

A biomarker is a molecule, such as a protein or gene, that is released by cancer cells or by the body in response to cancer. Clinically, it has served as an indicator of normal biological processes or pathological changes associated with disease progression, particularly in cancer. Previous research has focused on discovering key biomarkers across various areas of oncology. These biomarkers have been utilized as diagnostic tools for early detection, prognostic assessment, and predicting possible resistance to certain treatments. Biomarkers are valuable in modern medicine as they enable physicians to personalize therapeutic strategies according to the unique molecular characteristics of each patient [6].

Biomarkers in oncology can be classified into three distinct categories: diagnostic, prognostic, and predictive. Diagnostic biomarkers are employed for the detection of specific cancers and their subtypes. Moreover, they play a crucial role in cancer classifications. Historically, cancer classifications had been based on the anatomical site. Diagnostic biomarkers now enable clinicians and researchers to classify cancers according to their morphological features and microscopic characteristics [7]. Prognostic biomarkers help physicians anticipate the probable progression and behavior of cancer. Furthermore, they support physicians in estimating outcomes in specific patient populations, guiding treatment strategies aimed at improving the patient’s quality of life [8]. Predictive biomarkers are used to determine whether particular therapies are likely to benefit patients based on the biomarkers expressed in their tumors, therefore facilitating more individualized treatment approaches [9].

This study focuses on both clinically established and emerging molecular biomarkers in colorectal and gastric cancers. In colorectal cancer, the discussion will focus on KRAS, BRAF; carcinoembryonic antigen (CEA); and novel biomarkers such as circulating tumor DNA (ctDNA), methylated SEPT9, and selected microRNAs. For gastric cancer, the biomarkers of interest include HER2, PD-L1, and CA19-9, along with emerging biomarkers such as long non-coding RNAs. Each of these biomarkers is explored in terms of its diagnostic, prognostic, and predictive value, as well as its implications for precision medicine and targeted therapy.

## 2. Biomarkers in GI Cancers

### 2.1. Early Detection

Early detection is crucial in the field of oncology, and it is closely linked to better prognosis in cancer patients. Patients diagnosed earlier typically experience a better prognosis due to the increased effectiveness of treatment during the initial phases of disease progression. However, currently, there are no stand-alone biomarkers to detect and diagnose precancerous stages and early stages of CRC and gastric cancer due to their low sensitivity and specificity. As a result, recent studies have made an effort to focus on creating a panel combining different biomarkers to increase sensitivity and specificity, as well as discovering novel diagnostic biomarkers with higher sensitivity and specificity.

#### 2.1.1. Traditional Diagnostic Biomarkers for CRC-CEA and Other Glycoproteins

Carcinoembryonic antigen (CEA) is one of the most widely known conventional tumor markers for various types of cancer. However, CEA itself is not suitable for a standalone diagnostic biomarker for CRC due to a high rate of false positives [10]. Previous studies have demonstrated the limitations of CEA as a single diagnostic tool for CRC. However, they have also discovered that when CEA is combined with CA19-9, CA242, CA72-4, and CA125 as a panel for blood, it markedly increases sensitivity and specificity. When CEA is used alone for early-stage CRC detection, the sensitivity ranges from 18.8% to 52.2%, while panels with CEA and the other four glycoproteins show a sensitivity of 85.3% and a specificity of 95% for early-stage CRC detection [11].

Additionally, recent studies have demonstrated the potential of fecal CEA to be a more sensitive biomarker than serum CEA for early-stage and even precancerous CRC. It provides a significant option for patients who refuse colonoscopy by possibly offering them a non-invasive alternative with enhanced diagnostic accuracy [12].

Beyond early diagnosis, CEA also plays a role in detecting metastatic disease. Recent studies have demonstrated that combining serum CEA with MRI enhances the diagnostic accuracy for liver metastases in CRC patients. This underscores CEA’s value in both early detection and advanced disease monitoring [13]. These are especially significant findings for surgeons, as they are useful when planning surgery for CRC patients.

#### 2.1.2. Emerging Non-Invasive Blood-Based Biomarker for CRC-SEPT9

Given the limitations of CEA as a stand-alone diagnostic biomarker for CRC, recent research has focused on circulating tumor DNAs, which are released from tumor cells and circulate in the bloodstream [5]. Particularly, previous studies have demonstrated DNA methylation markers, like *SEPT9*, as promising non-invasive alternatives.

*SEPT9* is a set of DNA methylation markers currently used as an FDA-approved diagnostic biomarker for CRC in combination with *N-myc* downstream-regulated gene 4 (*NDRG4*) and bone morphogenetic protein 3 (*BMP3*) [14]. Among methylation-based biomarkers for CRC, *SEPT9* is considered one of the most clinically validated and widely used markers for CRC detection, particularly in non-invasive blood-based assays. Accordingly, the *SEPT9* test has been made commercially available as a CRC screening tool under names such as Epi proColon 2.0 and ColoVantage^®^ [15]. Its diagnostic performance—particularly sensitivity and specificity—has been extensively validated in prior studies. Studies by Wu et al. have demonstrated the possibility of the *SEPT9* gene methylation assay as a reliable tool for opportunistic CRC detection with a sensitivity of 76.6% and a specificity of 95.9% [16].

#### 2.1.3. Liquid Biopsy for CRC and Gastric Cancer

Traditionally, the diagnosis of CRC and gastric cancer is determined by imaging and pathological tissue biopsy [17,18]. However, several drawbacks are associated with imaging and tissue biopsy. First, tissue biopsy is invasive, and there is a risk of infection depending on the location from which the tissue sample is acquired. Moreover, sometimes, an insufficient number of tissue samples can be obtained, leading to unrepresentative results [18]. Therefore, there is a need to develop a non-invasive diagnostic technique to detect early CRC and gastric cancer.

**Figure 1:**
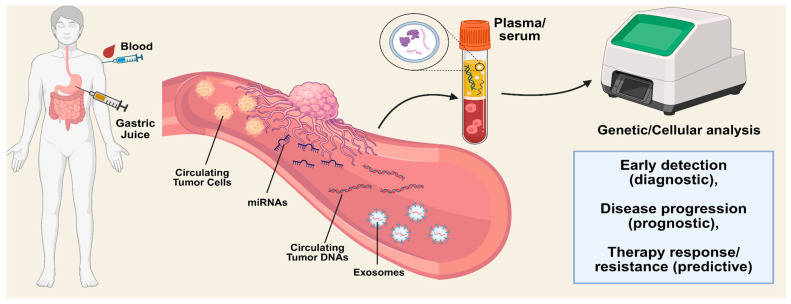
Overview of liquid biopsy for CRC and gastric cancer, Created in BioRender. Han, D. (2025) https://BioRender.com/hcs94c4. Blood samples, gastric secretions, and stool samples are collected for analysis. ctDNAs, CTCs, miRNAs, and exosomes are isolated from the liquid sample. Genetic and cellular analyses are used to detect biomarkers, and the results are interpreted.

Liquid biopsy is the emerging substitute for tissue biopsy. As shown in Figure 1, with liquid biopsy, circulating tumor cells (CTCs), exosomes, circulating tumor DNA (ctDNA), and tumor-educated platelets (TEPs) from body fluids, including saliva, urine, blood, and gastric juice, can be measured to detect CRC and gastric cancer [19].

#### 2.1.4. Novel Liquid Biopsy Associated Biomarkers for CRC–CTCs and ctDNAs

CTCs are motile cancer cells circulating in the bloodstream after being released from the primary tumor site [20]. Since CTCs are related to metastasis stages of cancers, they were initially thought to be only beneficial for late-stage diagnosis of cancers [21]. However, a previous study in 2016 demonstrated that CTCs are present in the early stages of cancer, meaning that intravasation of tumor cells happens in the early stages of cancer [22]. However, despite this finding, CTCs cannot be used as a practical diagnostic tool for the early detection of CRC due to their low sensitivity and low specificity [23]. One of the contributors determined by previous research is the liver. CTCs released from the colon travel to the hepatic portal veins, and the liver holds on to the CTCs traveling from the colon [24].

#### 2.1.5. Traditional Diagnostic Biomarkers for Gastric Cancer

CEA has also been used as one of the most widely known conventional diagnostic biomarkers for gastric cancer. However, similar to CRC, due to its low sensitivity, CEA has not been used as a stand-alone diagnostic tool for gastric cancer. Previous studies have discovered that non-cancerous conditions like inflammation, smoking, and infection could result in increased CEA, which then leads to false positives for gastric cancer [25]. Moreover, the fact that it has been used as a supportive diagnostic tool for both gastric cancer and CRC shows us that it is not specific to a certain type of cancer. Similarly, when CEA was combined with CA 125, CA-19-9, and CA 72-4 as a panel for blood testing, the sensitivity against the early detection of gastric cancer increased markedly [26].

#### 2.1.6. Novel Liquid-Biopsy-Associated Biomarkers for Gastric Cancer miRNAs

Non-coding RNAs (ncRNAs) are portions of RNAs that are not translated into proteins. Instead, they have a special role in regulating gene expression [27]. Particularly, microRNAs (miRNAs) are small noncoding RNAs that are known to regulate gene expression post-transcriptionally. They do this by binding to the 3′-untranslated region of target mRNAs. Previous study efforts focused on the functions of miRNAs and have discovered that miRNAs play crucial roles in normal cellular homeostasis and pathological processes [28]. Recent studies have discovered and studied various types of miRNAs involved in gastric cancer cells. A previous study in 2022 demonstrated that combined biomarkers, including miR-181, miR-652, and CA72-4, showed a sensitivity of 92.5% and a specificity of 86.8% for early gastric cancer detection [29]. This ultimately demonstrates that miRNA panels can potentially be non-invasive, more accurate diagnostic biomarkers for early gastric cancer detection.

### 2.2. Prognostic Biomarkers: Biomarkers That Indicate Disease Progression, Recurrence Risk, or Survival Outcomes

#### 2.2.1. Significance of Prognostic Biomarkers

Prognostic biomarkers predict the likely course and outcome of cancer. This outcome may include cancer recurrence, the probable course of cancer progression, the pattern of metastasis, and possibly death [30]. There are multiple benefits to using prognostic biomarkers in the field of oncology. First, it helps physicians and researchers to classify patients into low- and high-risk groups. Moreover, it helps physicians determine whether their patients require more aggressive treatments or conservative monitoring. Last but not least, predicting the likely course of cancer allows patients and physicians to plan long-term strategies.

#### 2.2.2. Traditional Prognostic Biomarkers for CRC

CEA has a dual role as a biomarker; it is a great diagnostic biomarker if combined with other glycoproteins, and it is also often used as a prognostic biomarker. Particularly, CEA is often used as a preoperative prognostic biomarker to predict the risk of recurrence of CRC after surgery for CRC. Studies in 2019 demonstrated that there is a significant association between the elevation of preoperative serum CEA and the elevated risk of systemic recurrence of distant CRC (58% higher) [31]. Moreover, they demonstrated that the elevation of CEA was also associated with a lower five-year disease-free survival rate [31]. Studies have also discovered that serum CEA is the most sensitive in predicting hepatic metastasis, as mentioned in this review earlier in the diagnostic biomarker section [31]. A previous study in 2023 showed that the elevation of the initial CEA level was associated with the recurrence of CRC, and the elevation of serum CEA at recurrence was associated with a lower survival rate in patients [32].

**Figure 2:**
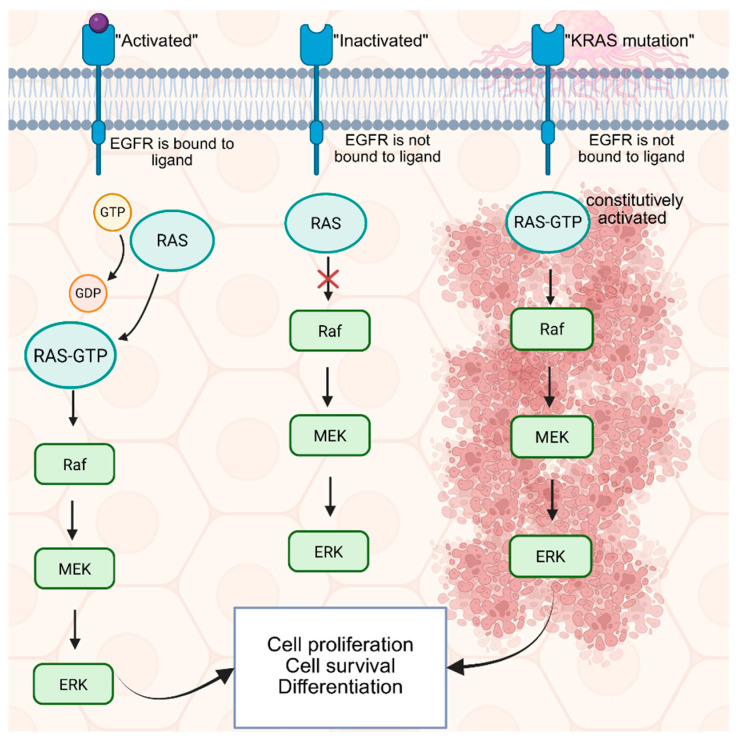
Overview of KRAS signaling under normal and mutated conditions, Created in BioRender. Han, D. (2025) https://BioRender.com/6ly19j1. In normal cells, when EGFR is bound to its ligand, it is phosphorylated. Then, it activates RAS with GTP. When RAS is bound to GTP, it activates the downstream MAPK pathway, leading to cell proliferation, survival, and differentiation. When RAS is mutated, RAS is bound to GTP independent of EGFR, and the MAPK pathway is constitutively activated, leading to tumor growth. Since RAS is activated independently of EGFR, it will result in resistance to anti-EGFR treatments.

*Kirsten rat sarcoma* (KRAS) is one of the most commonly found mutations in CRC. Previous studies have demonstrated that the KRAS mutation is reported in 40% of CRC patients [33]. As shown in Figure 2, the mechanism of KRAS is aberrantly activating the downstream RAS-RAF-MEK-ERK signaling cascade, which is a key player in regulating gene proliferation and differentiation and survival of the cell [34,35]. Although KRAS is one of the most widely known and studied predictive biomarkers, previous studies have shown that for metastatic CRC disease, patients with the KRAS mutation have a worse prognostic outcome than those with wild-type KRAS [34]. A previous study in 2014 demonstrated that ctDNA showed a sensitivity of 87.2% and a specificity of 99.2% for KRAS gene mutations, which is one of the biomarkers of CRC [36]. However, previous studies have also demonstrated that in stage II-III colon cancer, the KRAS mutation lacks significant prognostic relevance [37].

#### 2.2.3. Novel Prognostic Biomarkers for CRC

miRNAs are also emerging prognostic biomarkers for CRC. Recent studies have been focusing on discovering prognostic miRNAs to better predict the outcomes of CRC. A previous study demonstrated a higher expression rate for miR-155 and a significant association of high levels of miR-155 with advanced TNM stages, lymph node involvement, and distant metastasis [38]. That study emphasizes that miRNAs are potential prognostic biomarkers for CRC. A previous study in 2011 demonstrated that elevation of miR-375 and a low level of miR-142-5p significantly correlate with a higher recurrence rate and a poor survival rate [39]. Similarly, a study in 2012 demonstrated that elevation of miR-335 is also associated with a higher recurrence rate and a poor survival rate [40].

#### 2.2.4. Traditional Prognostic Biomarkers for Gastric Cancer

Cancer-related carbohydrate antigen 19-9 (CA19-9) is a significant prognostic biomarker for gastric cancer. A previous meta-analysis study in 2015 demonstrated that elevated CA19-9 levels were associated with poor disease-specific survival rate, disease-free survival rate, and worse overall survival rate in patients across multiple stages and tumor features [41]. This implies that patients with high CA 19-9 are often associated with more aggressive types of gastric cancer. CA19-9 is a valuable prognostic biomarker for late-stage gastric cancer. A recent study in 2020 demonstrated that preoperative CA19-9 is a valuable prognostic biomarker for patients with stage 3 gastric cancer [42]. Moreover, CA19-9 can also be utilized as a valuable biomarker to predict the risk of recurrence of gastric cancer after radical gastrectomy. A recent study in 2024 demonstrated that elevation of CA19-9 was associated with a lower 5-year survival rate after surgery [43].

#### 2.2.5. Novel Prognostic Biomarkers for CRC and Gastric Cancer

Despite their function as diagnostic biomarkers, CTCs are emerging as potential prognostic biomarkers for both CRC and gastric cancer. A study in 2014 demonstrated that CTCs and disseminated tumor cells (DTCs) are possible indicators for poor prognosis in gastric cancer patients, with evidence that there is a significant correlation between the detection of CTCs and gastric cancer prognosis [44]. Moreover, a previous study in 2015 demonstrated that the presence of CTCs was significantly associated with a shorter progression-free survival rate and overall survival rate [45]. A study in 2013 demonstrated that CTCs are a better predictor of the recurrence of stage 3 colon cancer than CEA. The presence of CTCs was significantly associated with lower disease-free and overall survival, emphasizing that CTCs are a better prognostic biomarker than CEA [46].

### 2.3. Predictive Biomarkers for Targeted Therapy: Biomarkers That Guide Treatment Selection

#### 2.3.1. Significance of Predictive Biomarkers

Predictive biomarkers are significant in the field of oncology. They provide information on whether patients with cancers with certain molecular characteristics benefit from a particular therapeutic intervention, including resection, chemotherapy, immunotherapy, and targeted therapy. Previous research efforts have focused on identifying these predictive biomarkers and determining their mechanisms and resistance to certain treatments.

#### 2.3.2. Traditional Predictive Biomarkers for CRC

The KRAS mutation abnormally activates the RAS-RAF-MEK-ERK signaling cascade, which is responsible for regulating proliferation, differentiation, and survival [34,35]. KRAS is a well-established predictive biomarker. The RAS-RAF-MEK-ERK signaling cascade is downstream of the epidermal growth factor receptor (EGFR). Therefore, patients with a KRAS mutation are resistant to anti-EGFR treatment as RAS-RAF-MEK-ERK will be persistently activated regardless of the activation of EGFR [47]. Although anti-EGFR treatment inhibits EGFR, the downstream RAS-RAF-MEK-ERK is constitutively active, leading to persistent tumor growth. Due to its resistance against anti-EGFR therapy and poor survival rate, developing a therapy against the KRAS mutation is crucial. Among the many KRAS variants, only the KRAS G12C allele has FDA-approved targeted therapies [48].

Similar to the KRAS mutation, the BRAF mutation also constitutively activates the MAP kinase pathway (RAS-RAF-MEK-ERK), leading to hyperproliferation. The BRAF mutation is reported in approximately 10% of CRC patients. A previous study in 2019 demonstrated that a RAS-independent non-V600 BRAF mutation (class 2) was unresponsive to anti-EGFR therapy, while a RAS-dependent BRAF mutation (class 3) was responsive to anti-EGFR treatment [49]. Among the patients with the BRAF mutation, 90% of the mutations are BRAF V600E. The BRAF V600E is known to have rapid progression and poor prognosis [50]. This demonstrates the urgency of developing therapies that target the BRAF V600E mutation. A recent study in 2021 demonstrated that a combined therapy of encorafenib (a kinase inhibitor) and cetuximab (an EGFR inhibitor) increased the overall survival rate, objective response rate, and progression-free survival rate in patients who were previously treated for BRAF V600E-mutated CRC in a metastatic setting [51].

#### 2.3.3. Novel Predictive Biomarkers for CRC

In addition to their function as diagnostic and prognostic biomarkers, miRNAs are emerging predictive biomarkers for both CRC and gastric cancer. Overexpression of miR-21 is associated with chemotherapy drug resistance, specifically 5-fluorouracil (5-FU) chemotherapy [52,53]. In fact, it has been demonstrated that combined therapy with 5-FU and miR-21 inhibitor via engineered exosomes has the potential to overcome drug resistance and improve the outcome of CRC treatments [53]. A previous study demonstrated that miR-1246, miR-135b, miR-1229, miR-96-5p, and miR-21-5p were elevated in exosomes from media that had chemoresistant cells [54]. Similarly, previous research has identified certain miRNAs as predictive biomarkers. A study in 2014 demonstrated that elevation of miRNA27a was significantly associated with a poor overall survival rate and that miRNA27a has the potential to predict resistance against fluoropyrimidine-based chemotherapy [55].

#### 2.3.4. Traditional Predictive Biomarkers for Gastric Cancer

Human epidermal growth factor receptor (HER2) is an upstream regulator of the MAPK pathway, and it plays a significant role in regulating cell differentiation, survival, and proliferation. HER2 forms homodimers with other HER2s or heterodimers with HER3 or EGFR. The type of dimerization differs depending on the cancer type. Dimerization of HER2 activates the downstream signaling cascade [56]. In HER2-mutated gastric cancer, HER2 can both homodimerize and heterodimerize with HER3 or EGFR [57]. When HER2 is constitutively activated regardless of the presence of the ligands, it leads to cancer. A previous study in 2010 demonstrated that combined treatment of trastuzumab with standard chemotherapy significantly increased the overall survival rate in patients with HER2-positive advanced gastric cancer [58]. Developing a second-line therapy for HER2-positive gastric cancer is still an ongoing issue. Recent studies have been focusing on discovering novel second-line treatments for HER2-positive gastric cancer for patients who have ineffective first-line treatments. In 2023, it was demonstrated that trastuzumab deruxtecan was a potential second-line treatment for HER2-positive gastric cancer with significant clinical improvement and an acceptable safety profile [59]. A study in 2022 demonstrated a significant objective response for ramucirumab plus paclitaxel in HER2-positive gastric cancer patients, but it did not result in an improvement in the overall survival rate [60].

#### 2.3.5. Novel Predictive Biomarkers for Gastric Cancer

Among many types of treatments for gastric cancer, immune checkpoint inhibitors, such as PD-L1 and PD-1 inhibitors, have recently emerged to treat advanced gastric cancer [61]. Programmed cell death-1 ligands (PD-L1) are expressed in the malignant cell membrane, and programmed cell death-1 (PD-1) receptors are present in immune T cells. The interaction between PD-L1 and PD-1 allows cancer cells to send inhibitory signals to T cells, thereby inhibiting the activity of T effector cells. Moreover, PD-L1 overexpression is often observed in various types of cancer. Recent studies have been focusing on targeting PD-L1 to find the optimal treatments for PD-L1-overexpressing gastric cancer patients [61]. A recent study in 2025 demonstrated that elevated BATF2 expression levels were associated with low PD-L1 expression. This emphasizes that BATF2 is a potential biomarker for predicting the efficacy of PD-1 and PD-L1 inhibitor treatments in gastric cancer patients [62].

## 3. Discussion

### 3.1. Comparative Analysis of Diagnostic Biomarkers-CRC

CEA and glycoproteins have been widely used as clinical diagnostic biomarkers. A previous study by Stollberg et al. demonstrated that among 36,537 patients, CEA was used for 43.9% of all tumor marker tests in the clinic, and glycoproteins like CA125, CA15-3, and CA19-9 were used 17.7%, 15.8%, and 14.9% of the time, respectively [63]. As mentioned earlier, mSEPT9 is currently commercially available, but it is not as widely available as CEA and glycoproteins. Previous studies have demonstrated the sensitivity and specificity of CEA, which are 59% and 89%, respectively [64]. Previous meta-analysis have demonstrated the sensitivity and specificity of *mSEPT9* as 71% and 92%, respectively. Despite CEA being widely used, previous studies have shown that it may not be ideal as an independent diagnostic tool due to its false positivity. The cost-effectiveness of mSEPT9 is higher than FIT and colonoscopy [65]. However, if a patient has a positive mSEPT9 result, he or she should be followed up with a colonoscopy in order to be diagnosed with CRC. Therefore, neither CEA nor mSEPT9 is ideal as an independent diagnostic biomarker for CRC.

### 3.2. Comparative Analysis of Diagnostic Biomarkers–Gastric Cancer

CA19-9 is available for gastric cancer, and it is commonly used in prognosis and monitoring [41]. In a previous meta-analysis of CA 19-9, studies demonstrated the sensitivity of CA 19-9 ranging from 6.8% to 51.7% [41]. They also demonstrated that higher sensitivity was correlated with higher stages of gastric cancer. A previous study by Wei et al. demonstrated the sensitivity and specificity of miRNAs for gastric cancer as 76% and 81%, respectively [66]. The advantages of CA19-9 include low cost and standardization, and its disadvantages include low sensitivity (false positives). The advantages of miRNAs include potential higher diagnostic accuracy when multiple miRNAs are combined, and their disadvantages include high cost due to multiple miRNA panels and a lack of assay standardization. Further studies are required for assay standardization and to test its validity and reproducibility as a gastric cancer screening tool.

### 3.3. Current Gold Standard for CRC Detection

The current gold-standard methods for CRC detection are colonoscopy and FIT. Despite colonoscopy being the gold-standard method, it is not convenient for certain patient populations. A previous study in 2019 demonstrated that there was a significant increase in post-colonoscopy complications in elderly populations and patients with inflammatory bowel disease (IBS) [67]. Moreover, the adenoma detection sensitivity can vary among practitioners. A study in 2022 highlighted that hospitals in rural or underserved areas may face constraints such as equipment or specialized training opportunities. This underscores the importance of ongoing professional development and support to maintain high-quality standards [68].

### 3.4. Current Gold Standard for Gastric Cancer Detection

Similarly, the current gold-standard method for gastric cancer detection is esophagogastroduodenoscopy (EGD), also known as upper GI endoscopy. Similar to colonoscopy, the advantages of EGD include direct visualization of the gastric mucosa and the ability to detect precancerous and early stages of gastric cancer.

### 3.5. Advantages of Liquid Biopsy

In contrast, the advantages of liquid biopsy include the following: 1. It is associated with many biomarkers that are higher in sensitivity and specificity than those of conventional biomarkers. 2. It is a non-invasive detection method that is also convenient for elderly patients and patients with IBS. 3. The biomarkers detected by liquid biopsy include all three types: diagnostic, prognostic, and predictive.

### 3.6. Limitations of Liquid Biopsy

Despite the advantages of liquid biopsy over colonoscopy, colonoscopy is still considered the gold standard for CRC detection. A recent study compared the cost-effectiveness of colonoscopy to liquid biopsy. The study demonstrated that colonoscopy is the most cost-effective detection method when compared with FIT, the Stool DNA test, liquid biopsy, and colonoscopy–liquid biopsy hybrid screening [69]. Moreover, if positive, FIT, the Stool DNA test, and liquid biopsy often require a follow-up colonoscopy, making the overall detection process even more expensive. Accordingly, liquid biopsy is not yet ready to serve as the new gold standard for CRC and gastric cancer detection.

## 4. Conclusions

There is an emerging transition regarding GI biomarkers toward non-invasive detection methods focused on improving the early detection rate, prognostic assessment, and personalized treatment with predictive biomarkers. Conventional biomarkers such as CEA and CA19-9 are still being utilized as practical diagnostic tools in the clinical monitoring of both CRC and gastric cancer. However, their insufficient sensitivity indicates the need for novel biomarkers with higher sensitivity. With the recent advances in the liquid biopsy method, novel biomarkers such as CTCs, ctDNA, methylated DNA assays (*SEPT9*), miRNAs, and exosomes have demonstrated increased sensitivity for the early detection of CRC and gastric cancer.

Traditional prognostic biomarkers such as CEA, CA19-9, and KRAS have been utilized to predict the likely course of disease progression in cancer, allowing physicians to personalize their patients’ treatments based on their cellular characteristics. There are emerging novel prognostic biomarkers, such as CTCs and miRNAs, providing more accurate predictions of possible outcomes of CRC and gastric cancer. Predictive biomarkers are one of the most essential biomarkers for CRC and gastric cancer treatment. KRAS, BRAF, PD-L1, and miRNAs determine whether certain populations of patients have resistance or sensitivity to certain therapies.

Previous studies have shown the limitations of single biomarkers, lacking sensitivity or specificity, and their decreased potential as biomarkers. This underscores the discovery of multi-marker panels, which may be the key to increasing sensitivity and specificity. For instance, as mentioned earlier, previous studies have shown some promising increases in sensitivity and specificity when multiple markers are combined [26,29]. However, there are multiple challenges in developing and validating these panels. There must be multiple studies performed in order to prove their validity and reliability across different patient groups. Moreover, the technical demands of multi-marker testing may limit accessibility. Also, the interpretation of multi-marker testing can be challenging due to potential variability, as well as a lack of standardization.

There are multiple limitations to liquid biopsy. First, the cost-effectiveness of liquid biopsy is a major issue that cannot be incorporated as a gold-standard technique for CRC and gastric cancer. Second, large-scale studies are required in order to test the validity and reproducibility of novel biomarkers. Lastly, although the novel biomarkers from liquid biopsy have shown higher sensitivity or specificity, the early detection rate is still being studied by many researchers. Future efforts should focus on optimizing the cost-effectiveness of liquid biopsy. There is also a need for simpler standardization to incorporate liquid biopsy in low-resource settings. AI-driven analysis could be the key to overcoming these limitations of liquid biopsy. AI tools can interpret results based on methylation patterns, nucleotide fragmentation patterns, and mutations, a task that requires significant time and effort if performed by humans. AI-driven analysis would efficiently decrease the time spent on data interpretation and streamline the overall diagnostic workflow by automating quality control and optimizing biomarker selection, thus reducing labor and operational costs.
